# Mechanisms by Which Liposomes Improve Inhaled Drug Delivery for Alveolar Diseases

**DOI:** 10.1002/anbr.202200106

**Published:** 2023-01-27

**Authors:** Laura T. Ferguson, Xiaonan Ma, Jacob W. Myerson, Jichuan Wu, Patrick M. Glassman, Marco E. Zamora, Elizabeth D. Hood, Michael Zaleski, Mengwen Shen, Eno-Obong Essien, Vladimir V. Shuvaev, Jacob S. Brenner

**Affiliations:** ^1^ Department of Medicine Pulmonary, Allergy, and Critical Care Division Perelman School of Medicine University of Pennsylvania Philadelphia PA 19104 USA; ^2^ Department of Systems Pharmacology and Translational Therapeutics Perelman School of Medicine University of Pennsylvania Philadelphia PA 19104 USA; ^3^ School of Biomedical Engineering, Science, and Health Systems Drexel University Philadelphia PA 19104 USA; ^4^ Emergency Medicine Department Yueyang Hospital of Integrated Traditional Chinese and Western Medicine Shanghai University of Traditional Chinese Medicine 200437 Shanghai China; ^5^ Department of Microbiology Perelman School of Medicine University of Pennsylvania Philadelphia PA 19104 USA; ^6^ Penn-CHOP Lung Biology Institute Perelman School of Medicine University of Pennsylvania Philadelphia PA 19104 USA

**Keywords:** inhaled, nanomedicine, nintedanib; pulmonary fibrosis, surfactant

## Abstract

Diseases of the pulmonary alveolus, such as pulmonary fibrosis, are leading causes of morbidity and mortality, but exceedingly few drugs are developed for them. A major reason for this gap is that after inhalation, drugs are quickly whisked away from alveoli due to their high perfusion. To solve this problem, the mechanisms by which nano‐scale drug carriers dramatically improve lung pharmacokinetics using an inhalable liposome formulation containing nintedanib, an antifibrotic for pulmonary fibrosis, are studied. Direct instillation of liposomes in murine lung increases nintedanib's total lung delivery over time by 8000‐fold and lung half life by tenfold, compared to oral nintedanib. Counterintuitively, it is shown that pulmonary surfactant neither lyses nor aggregates the liposomes. Instead, each lung compartment (alveolar fluid, alveolar leukocytes, and parenchyma) elutes liposomes over 24 h, likely serving as “drug depots.” After deposition in the surfactant layer, liposomes are transferred over 3–6 h to alveolar leukocytes (which take up a surprisingly minor 1–5% of total lung dose instilled) in a nonsaturable fashion. Further, all cell layers of the lung parenchyma take up liposomes. These and other mechanisms elucidated here should guide engineering of future inhaled nanomedicine for alveolar diseases.

## Graphical abstract

Inhaled drug‐loaded liposomes reach the distal lung where they interact with alveolar fluid, leukocytes, and parenchymal cells. The liposomes are not lysed or aggregated in bronchoalveolar lavage fluid, and alveolar macrophages are not saturable. Compared to oral dosing, these intratracheally instilled liposomes deliver 8000‐fold more cargo drug to the lung with a concomitant tenfold increase in lung half life.

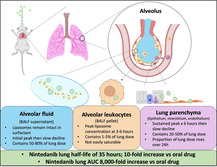

## 1 Introduction

Lung disease is an enormous burden, accounting for three of the top ten causes of mortality in the US in 2021, totaling >550 000 deaths (excluding lung cancer).^[^ ^1^ ^]^ The vast majority of these lung‐related deaths are due to diseases of the alveoli (air sacs), rather than disorders of the airways (conducting tubes, as in asthma). Alveolar diseases include the major killers of emphysema, pulmonary fibrosis, pneumonia, acute respiratory distress syndrome (ARDS), and, more recently, COVID‐19 pneumonia. Despite this huge burden, exceedingly few drugs have been approved for alveolar diseases.

The failure of drug development for alveolar diseases may seem surprising given that lung diseases have a very convenient delivery route: inhalation. Indeed, dozens of inhaled drugs have been developed for airways diseases. Aerosolized particles <5 μm in diameter will reach the airways and the distal alveolar tissue.^[^ ^2^ ^]^ However, for alveolar diseases, one of the major challenges is that after drugs deposit in the alveoli, they are rapidly whisked away into systemic circulation, due to the high perfusion rate of the alveolar tissue (>10× higher than most organs).^[^ ^3^, ^4^ ^]^ For example, the antifibrotic drug nintedanib is an oral drug that is efficacious in treating pulmonary fibrosis, but has severe dose‐limiting side effects outside the lungs.^[^ ^5^ ^]^ Inhaled nintedanib has been studied, but is so quickly whisked away from the lung and into systemic circulation that it has not supplanted oral delivery.^[^ ^6^, ^7^ ^]^

One method to utilize inhaled therapy for alveolar diseases is to use nanoscale drug carriers (nanocarriers).^[^ ^8^ ^]^ Nanocarriers generally offer two advantages: the ability to change a drug's PK properties, and the possibility of targeting a drug to a specific compartment or cell type. The potential of nanocarriers for lung disease was first illustrated clinically with nebulization (inhaled aerosol) of the antibiotic amikacin loaded into liposomes (Arikyace).^[^ ^9^, ^10^ ^]^ Arikyace gained FDA approval in 2018 for improving the treatment of nontuberculous mycobacteria, which primarily infects the airways.^[^ ^11^ ^]^ While this represents yet another triumph of therapy for diseases of the airways rather than the alveoli, it also suggests that alveolar diseases might similarly benefit from nanocarriers.

Unfortunately, the rational design of alveolar nanomedicine has been inhibited by our limited understanding of what happens to drugs and nanocarriers after they enter the lungs. While liposomes and other lipid‐based particles have been studied as agents for dry powder inhalation,^[^ ^12^ ^]^ gene delivery,^[^ ^2^, ^13^, ^14^ ^]^ and complement‐evading vehicles,^[^ ^2^ ^]^ few products have made it to market and many questions in inhaled nanomedicine are largely unanswered. After an inhaled drug‐loaded nanocarrier enters the lungs, how does the nanocarrier interact with surfactant? How much exits the extracellular space, and which cell types does it enter? Does it cross the epithelial barrier? Probing such questions will not only uncover new information, but also test commonly held assumptions. For example, based on nanoparticle‐antigen presenting cell interactions in the airways^[^ ^15^, ^16^, ^17^, ^18^, ^19^, ^20^ ^]^ and nanocarrier‐leukocyte interactions in the bloodstream,^[^ ^21^, ^22^ ^]^ it is often assumed that the vast majority of inhaled nanocarriers are rapidly taken up by alveolar macrophages, and that nanocarriers remaining in the alveoli will rapidly change their size and stability due to surfactant proteins.^[^ ^23^, ^24^, ^25^, ^26^, ^27^, ^28^ ^]^ It is also surmised that mucus and surfactant will impede nanocarrier delivery to the distal lung despite a growing body of in vitro literature demonstrating that nanocarrier properties such as increased PEG coating, protein coating, hydrophilicity, and neutral or negative charge improve passage through mucus and surfactant^[^ ^29^, ^30^, ^31^ ^]^ and in vitro and in vivo evidence that PEG may delay cellular uptake.^[^ ^32^ ^]^ Much of this work, however, is done in vitro and has not been quantitatively described using lipid‐based nanocarriers, which is the nanomaterial with by far the most clinically approved products (liposomes and RNA‐lipid nanoparticles). Testing these assumptions, and answering fundamental PK questions, will better guide the engineering of safer and more efficacious pulmonary therapies.

To probe these current gaps in understanding, here we have loaded a clinically used drug into nanocarriers for direct intra‐alveolar delivery by intratracheal instillation. Following nanocarrier delivery, we measured lung and systemic drug concentration and then correlated this measured PK to time courses of distribution among key lung compartments and cell types. As our test nanocarrier, we employed liposomes, the most clinically used nanoparticles with applications in cancer therapy and pulmonary targeting.^[^ ^2^, ^33^, ^34^, ^35^, ^36^ ^]^ Our test drug was nintedanib because it is commonly used for the lethal lung disease idiopathic pulmonary fibrosis, has burdensome side effects when given systemically, and disappointing lung PK when inhaled as a free drug (no nanocarrier).^[^ ^5^, ^6^ ^]^ As with prior inhaled nanocarriers,^[^ ^37^, ^38^, ^39^ ^]^ our nintedanib liposomes markedly improved lung PK; for example, liposome loading increased the area under the curve (AUC) of nintedanib concentration in the lungs by 35x compared to inhaled free drug.

To understand the mechanisms of this enormous improvement in lung PK, we then employed a wide array of techniques to quantify how the nanocarriers distributed over time to key lung compartments and cell types. We employed several techniques that are uncommon to studying inhaled nanomedicines: flow cytometry of disaggregated lung, after in vivo delivery, to quantify how well each cell type takes up nanocarriers; nanoparticle tracking analysis (NTA) to measure changes in nanocarrier size and concentration in the airspace over time; radiotracing of nanocarriers to compare nanocarrier distribution between the extracellular airspace (mucus + surfactant), airspace leukocytes, and lung parenchyma; and more common techniques such as liquid chromatography/mass spectrometry (LC/MS) and histology. Collectively, these new techniques provided results which challenge the above assumptions and suggest new conceptual models for lung PK of inhaled nanomedicine.

## 2 Results

### 2.1 Nintedanib‐Loaded Liposomes Have Advantageous Drug‐Loading Properties and Are Stable in Ex Vivo Bronchoalveolar Lavage Fluid

**Figure 1:**
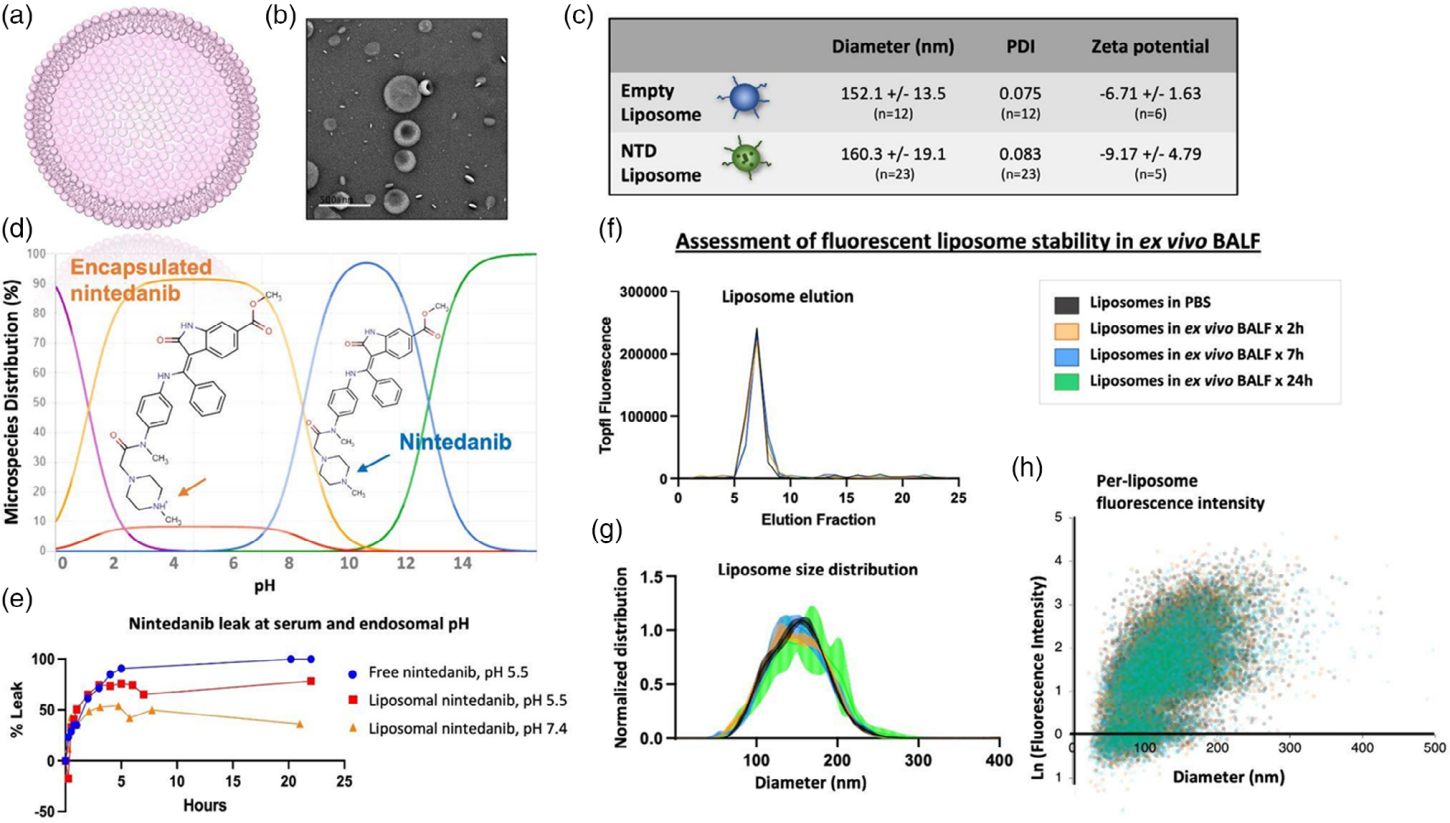
Nintedanib‐loaded liposomes have advantageous drug‐loading properties and are stable in ex vivo BALF. a) Schematic of liposome showing phospholipid bilayer with small molecule drug within the aqueous core. b) Negative stain electron microscopy of nintedanib‐loaded liposomes; scale bar, 500 nm. c) Hydrodynamic diameter, PDI, and zeta potential (mV) of empty and NTD‐loaded liposomes. d) Graph adapted from Chemicalize (Copyright 2022 Chemaxon) demonstrates nintedanib species at different pH. Shown in orange at pH 2–6, nintedanib's tertiary amine is protonated; this form is encapsulated in liposomes. Shown in blue at pH ≈9, nintedanib's tertiary amine is in a neutral state. e) Nintedanib leak out of liposomes over 24 h at varied pH shows less leak at neutral pH (as in serum and alveolar surfactant) versus acidic pH (as in endosomes), *n* = 1 preparation per pH. f) Assessment of fluorescent liposomes in ex vivo BALF up to 24 h, *n* = 1 per time point. Liposome elution by size exclusion chromatography shows no difference after incubation in BALF, and no free fluorophore peak. g,h) Assessment of fluorescent liposomes in ex vivo BALF up to 24 h, *n* = 5 technical replicates per time point. g) Size distribution and h) fluorescence intensity by size show complete overlap of all conditions, and therefore no difference after incubation in BALF. Error bars represent SEM.

We began by constructing PEGylated liposomes containing high concentrations of the FDA‐approved antifibrotic small molecule drug, nintedanib. Liposomes (**Figure** **1**a,b) were made using the thin film hydration method and loaded with nintedanib using remote loading, which leverages pH differences and salt gradient to trap charged nintedanib within the liposome core.^[^ ^40^ ^]^ Sucrose buffer solution was used to improve nintedanib solubility. We were able to reach an encapsulation efficiency of 28.5% ± 5.5 (*N* = 10) and a drug to lipid molar ratio of 0.2–0.35. These properties are well within industry standards for drug‐loaded liposomes.^[^ ^41^ ^]^ Liposomes maintained a consistent size, with only an 8 nm increase in diameter after drug loading (Figure 1c). Nintedanib leak out of the liposomes was tested using “infinite dilution dialysis” in a sucrose solution adjusted to the pH of plasma, and just above that of bronchoalveolar lavage fluid (BALF) (pH = 7.4), or that of endosomes (pH = 5.5) (Figure 1d). These studies demonstrated that nintedanib is more likely to leak out of liposomes in low pH such as in endosomes, rather than in serum or the alveolar airspace (Figure 1e).

The stability of fluorescent liposomes was quantified by measuring their size and fluorescence intensity after incubation for up to 24 h in BALF. Liposomes containing 1–2% TopFL‐PC lipid within their phospholipid bilayer were used as the test particle because TopFL is detectable by both plate reader and NTA. First, after size exclusion chromatography there is no detectable fluorophore in elution fractions outside those corresponding to liposomes, showing that the TopFL‐PC tracer does not leave the liposomes to form free micelles at an appreciable rate (Figure 1f). Second, in Figure 1g,h, we used NTA to quantify fluorescence intensity and size of individual liposomes, as measured by fluorescence detection. Results for liposomes incubated in BALF are superimposed over liposomes in PBS, with no significant difference in the distributions of sizes or fluorescence intensities of liposomes bearing fluorophore. Additionally, liposome size distribution measured by fluorescence detection is stable over time, noting a nonsignificant right‐shift in liposomes after 24 h in BALF. Non‐normalized size distribution is shown in Figure S1a, Supporting Information, showing that the number of liposomes bearing detectable quantities of fluorophore does not change over 24 h in BALF. Liposome fluorescence intensity is shown as a histogram (corresponding to Figure 1h) in Figure S1b, Supporting Information. Therefore, because size distribution by fluorescence detection, fluorescence intensity per liposome, and the number of liposomes bearing detectable quantities of fluorophore are unchanged, we can conclude that exposure to ex vivo BALF for up to 24 h does not cause significant change in liposome size (no lysis or aggregation) or loss of the liposomes’ fluorescent tracer.

### 2.2 Inhaled Nintedanib‐Loaded Liposomes Confer Massive Increases in Lung Half‐Life and AUC Compared to Inhaled or Oral Free Nintedanib

**Figure 2:**
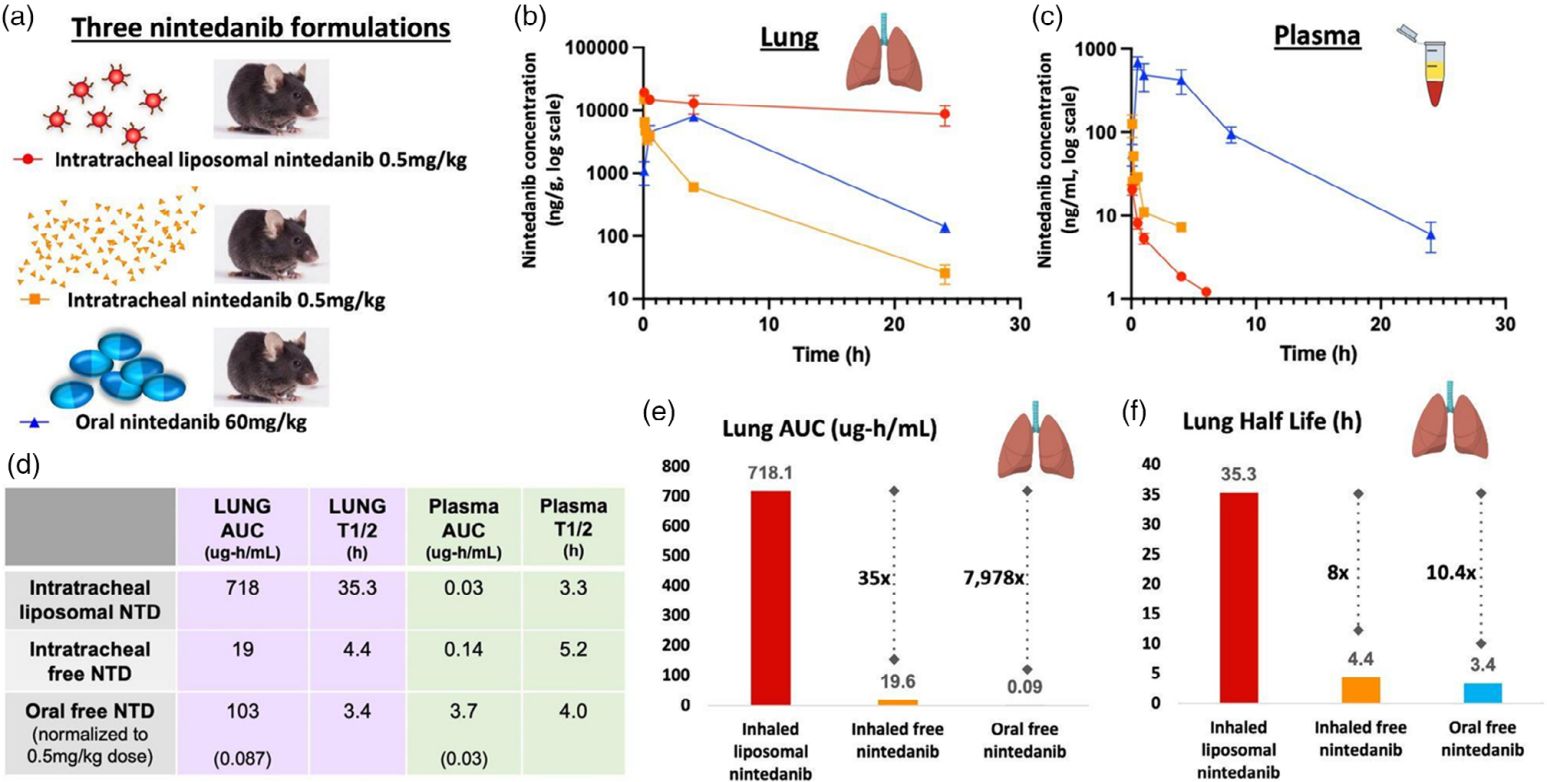
Inhaled nintedanib‐loaded liposomes confer massive increases in lung half‐life and AUC compared to inhaled or oral free nintedanib. Mice were given one of three nintedanib formulations, shown in a): intratracheal instillation of liposome‐loaded nintedanib (red), intratracheal instillation of free nintedanib (orange), or oral gavage of free nintedanib (blue; noting that clinically the drug is orally administered). Lung and plasma were harvested at time points up to 24 h, and then nintedanib concentration was measured by LC/MS. b,c) Nintedanib concentration over 24 h is shown for each formulation, measured by LC/MS; b) in the lung and c) in plasma. d) AUC and half‐life were calculated using GastroPlus noncompartmental modeling software from data obtained by LC/MS in panels (b) and (c). For oral dosing, AUC is shown for the dose given (60 mg kg^−1^) and then also normalized to the dose given by intratracheal administration (0.5 mg kg^−1^). e) Instilled liposomal nintedanib has a 35‐fold and 8000‐fold higher lung AUC compared to oral and instilled free drug (no nanocarrier), respectively. f) Instilled liposomal nintedanib has an eightfold and tenfold increase in lung half‐life compared to free instilled and oral drug, respectively. *N* = 3, error bars = SEM.

Having devised liposomes that encapsulate nintedanib with characteristics suitable for in vivo use (characterization, Figure 1; electron micrograph, Figure 1b), liposomal nintedanib was administered to mice via intratracheal instillation and compared to oral free nintedanib (“free” means no drug carrier), the current standard of care for pulmonary fibrosis,^[^ ^5^ ^]^ and intratracheally instilled free nintedanib (**Figure** **2**a). Importantly, the dose of inhaled drug was scaled down from that of oral drug based on known bioavailability of <5% after oral intake.^[^ ^42^ ^]^ Using liquid chromatography/mass spectrometry (LC/MS), we determined that liposomal nintedanib has superior lung PK compared to the other two formulations (Figure 2b–d, linear time scale and Figure S2, Supporting Information, log time scale). As shown, liposomal nintedanib remains in the lung at a high concentration for much longer than either delivery method with free drug while plasma concentration falls fastest for liposomal nintedanib. Notably, high plasma concentration or mucosal damage from direct gastrointestinal exposure is responsible for the severe side effects of oral nintedanib, especially dose‐limiting diarrhea.^[^ ^5^, ^43^ ^]^ Together, these observations on lung and plasma concentrations suggest that the therapeutic action of instilled liposomal nintedanib in the lung will be maximized while systemic off‐target side effects will be minimized. We then used GastroPlus to calculate the AUC and half‐life for each formulation using standard noncompartmental analysis (Figure 2d–f). We found that instilled liposomal nintedanib has an astounding 8000‐fold higher lung AUC and 10x increased lung half‐life versus oral drug (the current standard of care) and a 35‐fold higher lung AUC and 8x increased lung half‐life compared to instilled free drug. In summary, instilled liposomal nintedanib provides significantly better lung exposure to drug, with simultaneously less systemic exposure, compared to the current standard of care for nintedanib.

### 2.3 Liposomes Remain Intact and Do Not Aggregate Following Intratracheal Instillation

**Figure 3:**
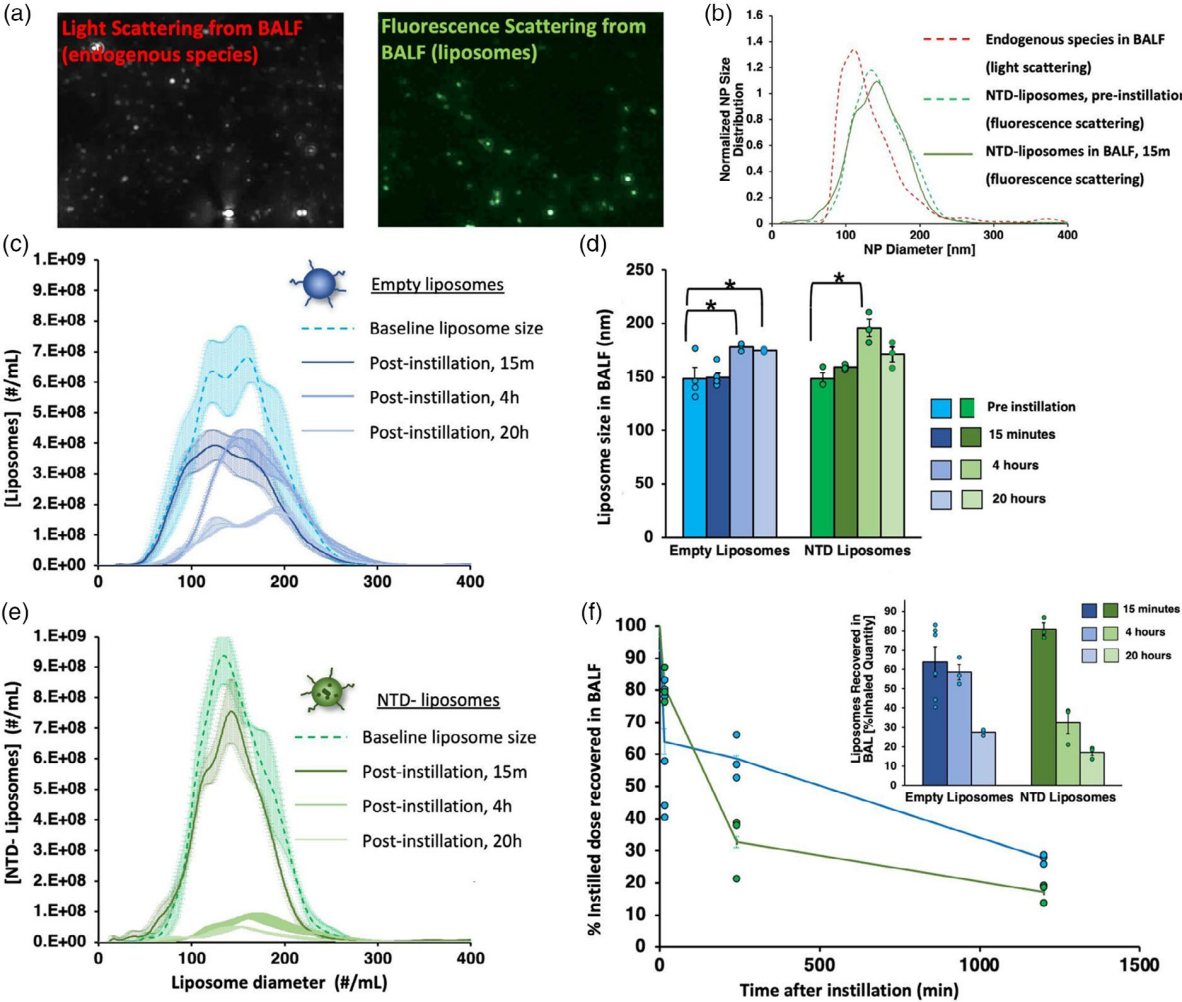
Liposomes remain intact and do not aggregate following intratracheal instillation. Mice were given 10 mg kg^−1^ lipid of fluorescent liposomes or nintedanib‐loaded fluorescent liposomes via intratracheal instillation, and then BALF was harvested and analyzed by NTA. a) Example primary data showing NTA of endogenous particles in BALF by light scattering (left) and liposomes in BAL by fluorescence scattering (right). b) Normalized histogram of nintedanib‐loaded liposomes in their native form (before being given to mice, dotted green line, fluorescence emission), compared to nintedanib‐loaded liposomes present in BALF 15 min after instillation (solid green line, fluorescence scattering), compared to endogenous particles + nintedanib‐loaded liposomes present in BALF 15 min after instillation (dotted red line). Nintedanib liposomes have the same size distribution before and after lung delivery. c–e) Size distribution of c) BAL‐extracted empty liposomes and e) nintedanib liposomes at 15 min, 4, and 20 h after liposome instillation, each compared to their preinstillation size distribution. d) Average size of liposomes over time [data from histograms in (c) and (e)], noting small size increase with increased time the liposomes have dwelled in the BALF layer of the lungs in vivo. f) Liposomes recovered in BAL over time, as a percentage of initial dose, showing that at least ≈20–30% of liposomes are recoverable intact in BALF as long as 20 h after instillation; inset is the same data depicted as averages. *N* = 3–6 for empty liposomes; *N* = 3 for nintedanib liposomes; error bars = SEM.

Having shown that intratracheally instilled liposomal nintedanib confers huge PK advantages, we next sought to elucidate the mechanisms behind these advantages. In particular, we wanted to understand the behavior of liposomes in pulmonary surfactant. If pulmonary surfactant quickly lyses liposomes, the PK advantage of inhaled liposomes would require mechanisms that take place after lysis. To assess this, we used NTA to measure the number, size, and fluorescence intensity of liposomes present in BALF collected from mice that received instilled liposomes 15 min, 4 h, or 20 h prior. As shown in **Figure** **3**a, NTA was performed in two modes: light scattering was used to count both endogenous spherical species (i.e., micelles and other lipid‐based conglomerates) plus liposomes, and fluorescence emission was used to detect exogenous fluorescently labeled liposomes only. Figure 3b shows a normalized size distribution after NTA detection and demonstrates two important points: first, that endogenous species (red line) have a smaller average diameter than liposomes; and second, that for at least 15 min after instillation, liposomes retain their diameter and size distribution. Video NTA data and additional primary data are shown in Figure S3, Supporting Information.

Next, we investigated the time course of changes in liposome size and concentration after instillation. Employing just fluorescence emission to detect only exogenous liposomes, we measured the diameter and concentration of both empty liposomes (blue, Figure 3c,d,f) and nintedanib (NTD)‐loaded liposomes (green, Figure 3e,d,f). Both liposome formulations remained present in BALF with a relatively stable diameter over the course of 20 h, although drug‐loaded liposomes had slightly more size variability (Figure 3c,e show size distribution and relative liposome count; Figure 3d shows size with SEM). Both formulations demonstrated significant retention in BAL at 15 min (64% retention for empty liposomes and 81% retention for NTD liposomes, Figure 3f). This retention fell over time, with both having 20–30% retention at 20 h. Notably, NTA analysis does not account for liposomes that have been taken up by alveolar leukocytes (the diameter of leukocytes is too large to be detected in this analysis), directly taken up by the lung parenchyma, or any initial dose that was inadvertently aspirated into the stomach. In summary, we demonstrated, based on particle size and size distribution, that instilled liposomes were neither broken down nor aggregated after in vivo instillation for up to 20 h or ex vivo exposure to BALF.

### 2.4 All Lung Compartments Contain Liposomes for At Least 24 h; Over Time, the Lung Parenchyma's Share of Total Lung Dose Increases, and Over Dose Range, Macrophages Do Not Demonstrate Saturability

**Figure 4:**
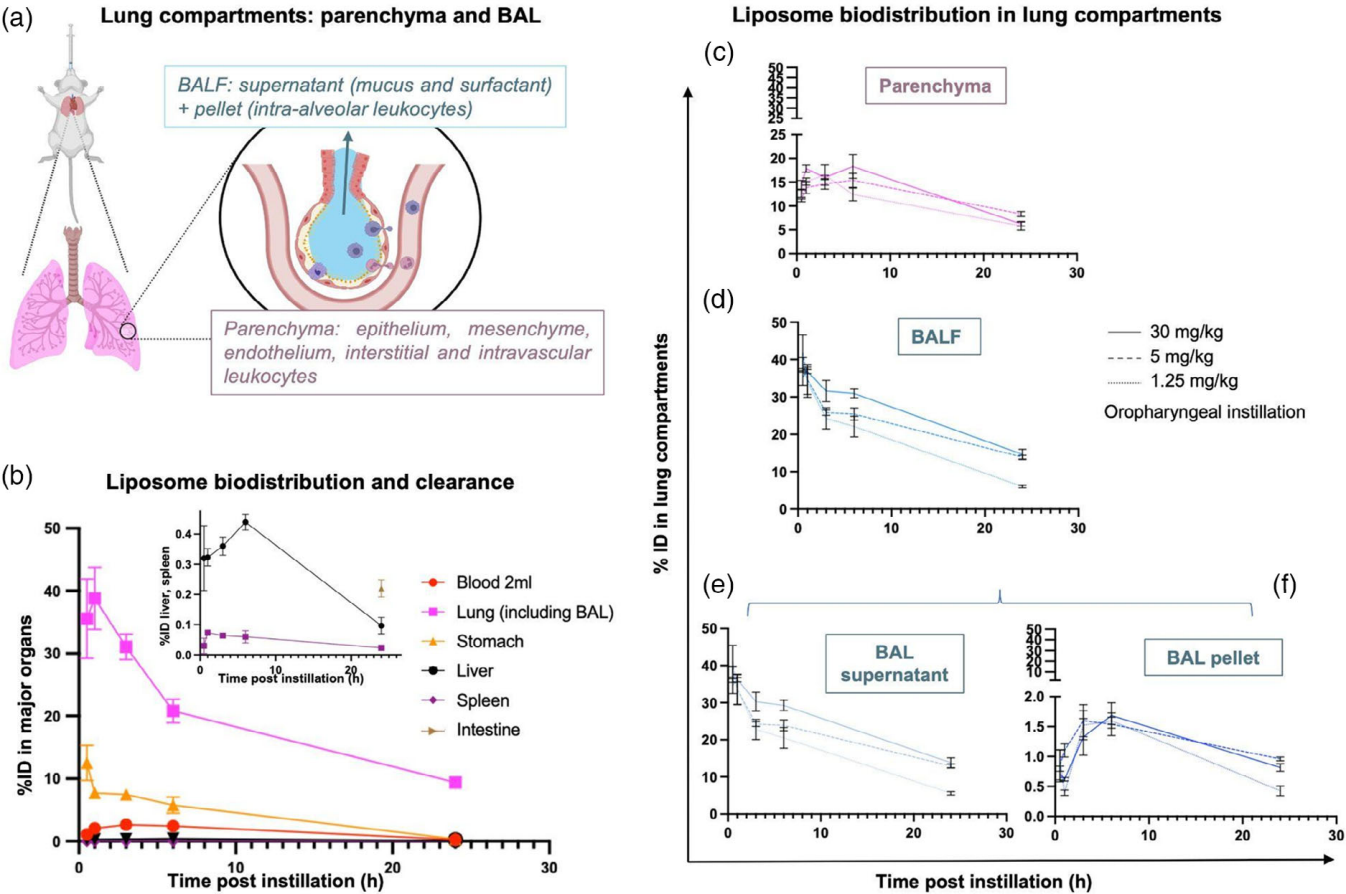
Inhaled liposomes behavior in BALF, alveolar macrophages, and lung parenchyma: over time, lung parenchyma's share of total lung dose increases; over dose range, macrophages do not demonstrate saturability. Mice were given liposomes labeled with ^125^I radiotracer via intratracheal instillation, then BALF was collected and organs were harvested at various time points as shown. Liposome localization was measured by a gamma counter. a) Schematic demonstrating intratracheal delivery into lungs with division into two initial compartments: the intra‐alveolar compartment (blue), consisting of liposomes taken up by intra‐alveolar leukocytes or suspended in the cell‐free BALF layer; and the parenchymal compartment (pink), which is composed of all lung cells except intra‐alveolar leukocytes. b) Biodistribution over time in blood, lung, stomach, and, in inset graph due to low total levels, in liver, spleen, and intestine. Lipid dose: 2.5 mg kg^−1^. c–f) Saturability study with varied lipid dose from 1.25 to 30 mg kg^−1^. Lung compartment biodistribution is divided into c) lung parenchyma, d) total BALF, e) BAL supernatant, and f) BAL pellet. *N* = 3, error bars = SEM.

Knowing that a significant portion of inhaled liposomes remain intact in the alveolar space through at least 20 h when given by intratracheal instillation, we next used radiotracing to quantify the fraction of inhaled liposomes that deposit in each key lung compartment over time. The lung was divided into two compartments (**Figure** **4**a): first, the lung parenchyma (all lung cells except for airspace leukocytes) and second, the intra‐alveolar space (referred to as BALF). This alveolar space is further divided into alveolar leukocytes and cell‐free BALF. To measure deposition in each of these compartments, radiolabeled liposomes were given via intratracheal instillation, and then a biodistribution of major organs was performed at time points up to 24 h (Figure 4b). Peak lung concentration was observed at 1 h, and at all time points the lung contained the largest fraction of instilled dose compared to other organs. Percent recovery, defined as the sum of collected organs over total administered dose, started at 50% and declined over time (Figure S4, Supporting Information). A portion of the initial dose was lost to tissues not harvested and therefore not quantified: the roof of oropharynx, esophagus, and small intestine. Additionally, any dose that entered the bloodstream (a small fraction, Figure 4b) also had the opportunity to diffusely deposit in all tissues at low concentrations. Notably, the blood and clearance organs (Figure 4b, inset) received only a fraction of liposome dose.

These biodistribution experiments included examination of BALF to sample liposome distribution in two key compartments: 1) airspace leukocytes, presumably macrophages,^[^ ^19^ ^]^ in the BALF pellet and 2) cell‐free pulmonary surfactant and mucus in the BALF supernatant (Figure 4c–f). As shown in Figure 4c,d, liposomes were initially observed in greater numbers in BALF compared to lung parenchyma, but over time more quickly left the BALF compartment. This represents either a shift from BALF into parenchyma, or a slower excretion from parenchyma. The only compartment with a clear increase in liposome accumulation after initial deposition is the alveolar macrophage compartment. Alveolar macrophages continue to gain liposomes over the first 4–6 h (Figure 4f) followed by a slow decline. To understand the ability of alveolar macrophages to take up liposomes, the instilled dose was varied from 1.25 to 30 mg kg^−1^, a liposome dose in vast excess of tha*t* tested in PK studies in Figure 2 or that needed to provide superior lung delivery compared to oral dosing or free drug instillation. At each dose, liposome uptake by alveolar macrophages is linearly related to initial instilled dose; at up to 30 mg kg^−1^ liposome dose, saturability was not observed (Figure S5, Supporting Information). Further probing the relationship between lung compartments shows that while alveolar macrophages are not saturable, they contain only 1–5% of the total lung liposome uptake. Alveolar fluid, conversely, contains 50–80% of total lung liposome uptake (decreasing over time) and lung parenchyma contains 20–50% of total lung liposome uptake (increasing over time).

In summary, Figure 3 and 4 collectively show that instilled liposomes survive intact for a prolonged period in BALF. Within the BALF, the alveolar macrophage compartment contains a minor fraction of instilled liposome dose but is not easily saturable. While the parenchyma contains fewer total liposomes than BALF, over time it is the only lung compartment to increase its fraction of total lung dose. Finally, both the alveolar leukocytes and lung parenchyma also slowly elute the liposomes’ tracer, thus suggesting that these compartments may act as “drug depots,” slowly (over hours) eluting drug to adjacent cells (e.g., epithelial cells eluting to mesenchymal cells).

### 2.5 Inhaled Liposomes Home to Both Alveolar Leukocytes and Lung Parenchymal Cells

**Figure 5:**
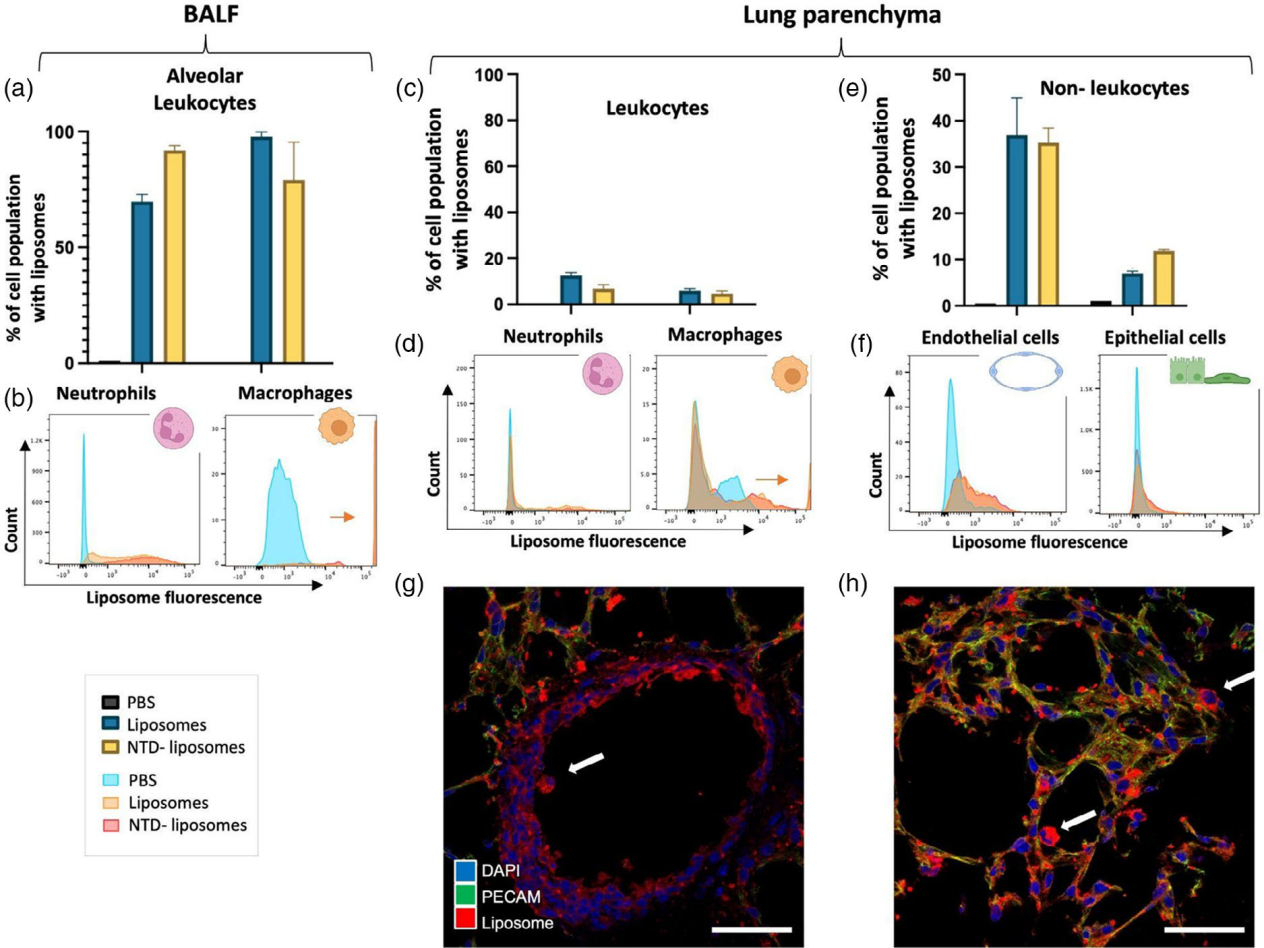
Inhaled liposomes home to both alveolar leukocytes and lung parenchymal cells. Mice were given fluorescent liposomes (empty and drug‐loaded) via intratracheal instillation, and then at 20 h whole lung was analyzed. a–f) Prior to harvest and preparation of single‐cell suspension for flow cytometry, BAL and perfusion were performed. a,b) Greater than 75% of alveolar leukocyte populations were associated with liposomes, but only macrophages (B, right panel) demonstrated a large shift in fluorescence, indicating significant uptake. c,d) Comparatively, parenchymal leukocytes (those embedded in the lung parenchyma, or at least firmly attached) were significantly less associated with liposomes (<15%), but parenchymal macrophages still demonstrated a significant shift (D, right panel). e,f) Cells of the lung parenchyma, such as epithelial cells and endothelial cells, were approximately 10% and 35% associated with liposomes, respectively, though with a much smaller shift in fluorescence compared to macrophages. g,h) IHC demonstrates liposomes (red) in all layers of lung tissue in both h) alveolated tissue and g) conducting airways. Airspace leukocytes are seen with significant liposome uptake (arrows). Scale bar = 50 μm. *N* = 2, error bars = SEM.

Having examined the above three large compartments of the lungs, we next endeavored to examine how inhaled liposomes distribute between different cell types. Mice were given fluorescent liposomes (empty and drug‐loaded) via intratracheal instillation, then at 20 h whole lung was disaggregated into a single‐cell suspension and analyzed by flow cytometry (**Figure** **5**a–f). Analysis was performed at 20 h, and post‐BAL, to minimize surreptitious binding between residual liposomes in the alveolar space and lung cells during single‐cell preparation. Our gating strategy is shown in Figure S6, Supporting Information. We found that at least 75% of alveolar leukocytes (neutrophils and macrophages harvested from the BAL) (Figure 5a) contained liposomes and that this was true for empty and nintedanib‐loaded liposomes. Importantly, while both types of leukocytes had liposomes, macrophages took up orders of magnitude more liposomes than neutrophils, demonstrated by the enhanced right‐shift population in Figure 5b (arrow). While macrophages take up a significant amount of liposomes on a per‐cell basis, it should be remembered that the BAL leukocytes as a whole take up no more than 2% of the total instilled dose (Figure 4f).

In addition to alveolar leukocytes, cells of the lung parenchyma (interstitial and marginated leukocytes, endothelial cells, epithelial cells, mesenchymal cells) were analyzed for liposome uptake. Analysis of parenchymal leukocytes (those residing deep to the alveolar space, Figure 5c,d) showed that only a minority of both neutrophils and macrophages took up liposomes, although macrophages again had a significant population shift indicative of greater uptake. Lung epithelial and endothelial cells (Figure 5e,f) showed that 35% of endothelial cells and 10% of epithelial cells took up liposomes. This significant endothelial uptake indicates that liposomes reach all cell layers of the lung. There were no significant differences between nintedanib‐loaded and empty liposomes with respect to cell targeting.

Immunohistochemistry (IHC) corroborates the findings elucidated by flow cytometry (Figure 5g,h; control with no liposomes is shown in Figure S7, Supporting Information). Liposome staining is seen in all layers of both distal alveolated lung tissue (Figure 5h) and proximal conducting airways (Figure 5g). Leukocytes—particularly airway leukocytes residing within the alveoli and the conducting airways (arrows)—are highly stained by liposomes.

### 2.6 Intratracheally Delivered Liposomes and Nintedanib Liposomes Are Nontoxic In Vivo

**Figure 6:**
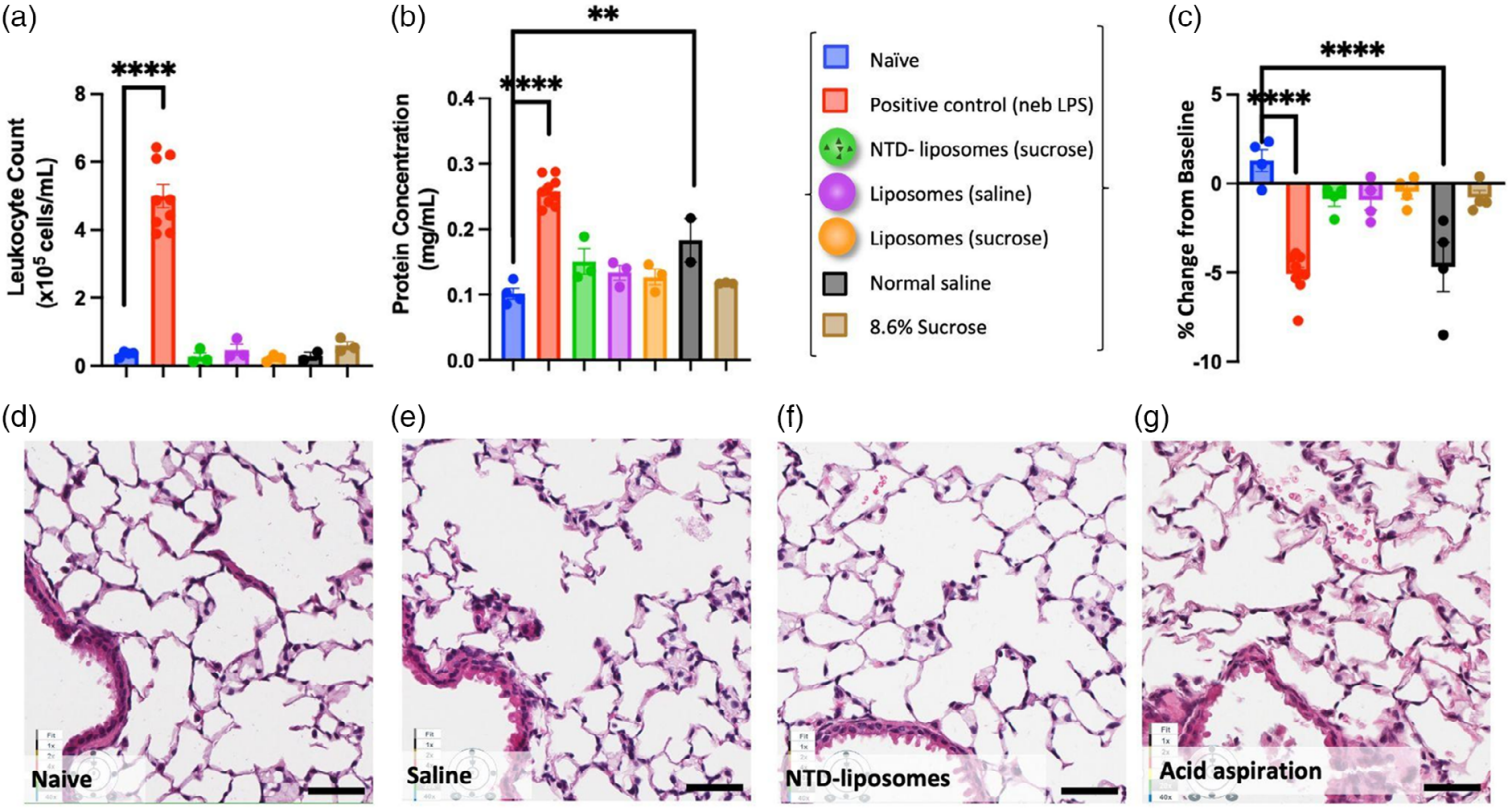
Intratracheally delivered liposomes and nintedanib liposomes are nontoxic in vivo. Toxicity studies were performed at 24 h. Naive murine lung is compared to nebulized LPS (a positive control for injury), intratracheal instillation of nintedanib‐loaded liposomes (0.5 mg kg^−1^ drug and 0.73 mg kg^−1^ lipid) in sucrose, empty liposomes (2.5 mg kg^−1^ lipid) in saline, empty liposomes (2.5 mg kg^−1^ lipid) in sucrose, saline buffer, or sucrose buffer. a) BALF leukocyte count; b) BALF protein concentration; c) weight change from baseline. *N* = 3 mice per group, one‐way ANOVA; **** *p* <= 0.0001, ** *p* <= 0.01. d–g) H&E histology shows no observable toxicity from intratracheal liposomes compared to acid instillation positive control; representative images, *n* = 3 mice per group with the exception of acid instillation, *n* = 1 and saline instillation, *n* = 2.

Using intratracheal instillation to approximate inhaled liposome delivery, we examined the tolerability of nintedanib liposomes in naive mice by measuring weight change as an indication of general health, BALF leukocyte and protein counts as indicators of inflammatory activity and endothelial leakage, respectively, and histology to observe potential perturbation of tissue architecture in the lung. All assays were obtained 24 h after intratracheal instillation of liposomes or controls. As a positive control, we subjected mice to either nebulized LPS chamber (for BALF toxicity readouts) or intratracheal instillation of 50 μL of 0.1 *N* hydrochloric acid (for histology assessment), which are both common models of acute lung injury.^[^ ^44^ ^]^ As shown in **Figure** **6**a–c, there were no statistically significant differences between mice that received liposomes versus naive controls in weight loss, BALF protein, or BALF leukocyte counts. Similarly, histology showed no detectable increase in red blood cell (RBC) aggregation, leukocytes infiltration into alveoli, or obvious tissue architecture damage, as shown by representative images (Figure 6d–g). Instillation of saline was noted to cause inflammation (BALF protein elevation) and weight loss. While saline is generally considered an innocuous carrier solution for inhalation in humans, it is known to produce a mild and transient inflammation in rodents.^[^ ^45^ ^]^ Nevertheless, we used a sucrose solution, which induced no inflammation, as a carrier solution for nintedanib liposomes in this study. Additionally, complete blood counts (CBC) were measured from each group. Instilled liposomes resulted in no change in leukocyte or platelet counts, and only slight increase in hematocrit (Figure S8, Supporting Information). Taken together, intratracheal instillation of drug‐loaded liposomes appears safe, resulting in no increase in lung or systemic inflammation as measured by BAL, CBC, and histology.

## 3 Discussion

We set out here to identify the mechanisms by which inhaled nanoparticles distribute to defined compartments and cells within the lungs after inhaled delivery, an area of active multidisciplinary research.^[^ ^3^, ^23^, ^24^, ^26^, ^28^, ^40^, ^46^, ^47^, ^48^ ^]^ Employing a variety of techniques not widely used in the inhaled field, a test drug that treats pulmonary fibrosis, and intratracheal instillation to approximate inhaled delivery, we demonstrated a number of findings that will help guide the engineering of inhaled nanomedicine.

First, we found that drug‐loaded liposomes remain intact and unaggregated for several hours after exposure to the intra‐alveolar space in vivo. Given that many surfactants lyse liposomes, and that agglutination of nanoparticles is common in other bodily fluids (e.g., serum), it may seem counterintuitive that pulmonary surfactant does not lyse or aggregate liposomes. However, our findings fit with an in vitro study from decades ago^[^ ^49^ ^]^ and an in vivo study from earlier this year.^[^ ^50^ ^]^ The measurements taken by NTA in Figure 3 likely underrepresent aggregated liposomes due to BALF dilution; nevertheless, we recovered 60–80% of the instilled liposomes at early time points and saw no aggregation, indicating that there was no aggregation in a sample representing the majority of the instilled liposome dose. Additionally, aggregation that is reversible by simple dilution likely does not affect performance of the particle because 1) this type of aggregation would not influence drug depot function, and 2) transiently aggregated liposomes will eventually be diluted as part of transit through and clearance from the body. The reproducibility of liposome diameter over time (when fewer and fewer liposomes remain in BALF and are thus naturally less concentrated) suggests that these results are valid. Thus, inhaled liposomes and other lipid nanoparticles are likely compatible with pulmonary surfactant and can be further developed for clinical use in alveolar diseases such as pulmonary fibrosis.

The second surprising finding is that alveolar macrophages do not dominate the uptake of inhaled liposomes. Tracking radiolabeled liposomes results in the clearest evidence for this claim. As shown in Figure 4f, the fraction of liposomes in BAL leukocytes (primarily alveolar macrophages) is only a tiny portion of the overall mass of lung liposomes. Specifically, at all time points, BALF leukocytes contain only 0.5–1.5% of the instilled dose of liposomes, orders of magnitude less than BALF supernatant (surfactant + mucus layer) and lung parenchymal cells. Further investigation of the alveolar macrophage compartment (Figure 4f, and Figure S5, Supporting Information) shows that macrophages do not saturate at a 24‐fold range of instilled dose. While not saturable, alveolar macrophages (Figure 5b) each take up far more liposomes than other cell types. Because there is only ≈1 macrophage for every three alveoli,^[^ ^51^ ^]^ the alveolar macrophage system as a whole has a minority of lung uptake, even though macrophages avidly take up liposomes, and therefore does not dominate liposome distribution after instillation.

Third, our results suggest that the lung compartments that initially receive the highest fraction of nanoparticles may serve as “drug depots,” eluting out the cargo drugs to nearby compartments or cell types. Figure 4 shows that all analyzed lung compartments slowly elute cargo, and that the lung parenchyma is the only compartment that increases its relative proportion of total lung dose over 24 h. The lung parenchyma remains relatively stable at early timepoints, while alveolar fluid (BAL supernatant) most rapidly elutes cargo in the first 6 h, suggesting that even though the alveolar fluid layer at its thickest is <1 μm,^[^ ^52^ ^]^ not all cargo is simply transferred to the parenchyma. Further, it suggests that for small molecule drugs, even lung parenchymal cell types that do not initially receive strong uptake on their own can receive significant drug delivery at later time points from drug eluted from nearby compartments and cell types.

In addition to the above insights into the mechanisms of inhaled nanomedicine, this study also describes a potentially clinically useful product, inhaled liposomal nintedanib. We show that liposomal nintedanib provides a massive increase in nintedanib delivery to the lung (increased lung AUC of 8000‐fold over oral delivery) with a concomitant reduction in plasma, with important potential clinical benefits. Nintedanib, prescribed for pulmonary fibrosis, has moderate efficacy in reducing the decline in lung function but has ample room for both improved efficacy and reduced side effects.^[^ ^5^, ^53^ ^]^ Our liposomal formulation has the potential to enable both of those outcomes. The results presented here will have to be tested in larger animals and with conventional nebulization, rather than intratracheal instillation, which allows a small but variable fraction of drug‐loaded liposomes to be delivered to the GI tract (Figure 4b). We anticipate an important aspect of this work will be to further refine the delivery system for use with a nebulizer as is done with other inhaled liposomal pulmonary drugs.^[^ ^9^ ^]^

The data presented here provide information about the biodistribution, PK, and behavior in BALF of instilled drug‐loaded liposomes but leaves three areas in particular open to further characterization. First, we hypothesize that nebulized delivery, essentially a mist of nanoparticles suspended in micrometer‐sized water droplets delivered to the distal lung, would spread to form a thin film not dissimilar to the bolus of liquid delivered by intratracheal instillation. This hypothesis should ultimately be tested using methods similar to that described here. Second, we did not characterize the behavior of liposomes in proximal conducting airways (coated in mucus) versus distal gas‐exchange airways (coated with surfactant), and instead used BALF. Therefore, we cannot yet describe contributions by proximal (conducting airways) versus distal (alveolar) lung tissue in driving the liposome behavior (Figure 3 and 4), or the drug behavior (Figure 2); however, IHC (Figure 5) reveals that liposomes do in fact reach both the proximal airways and distal alveolated lung tissue. It is therefore reasonable to assume—based on the massive surface area of the distal alveolar epithelium versus proximal epithelium—that liposome behavior in the alveolus drives the PK observed here. Third, in addition to further studies comparing airways versus alveoli, an additional area for future study is extending techniques to measure the cargo drug (nintedanib or other) in lung subcompartments, in addition to the nanocarrier (liposomes).

Inhaled liposomes, surviving in surfactant, optimize lung dose. We show an 8000‐fold improvement in lung AUC using instilled liposomal nintedanib compared to oral free drug. We have demonstrated liposome stability in BALF over time, and we have raised the interesting hypothesis that alveolar macrophages are not easily saturated and not the primary driver of prolonged drug release. These results, taken together, show great promise for improving therapeutics for chronic lung disease like pulmonary fibrosis, and provide guidance for engineering future therapeutics

## 4 Experimental Section

4.1

4.1.1

##### Liposome Synthesis and Characterization

Unilamellar liposomes were prepared as previously described^[^ ^54^, ^55^, ^56^ ^]^ with composition (mole%) 54% DSPC (1,2‐distearoyl‐sn‐glycero‐3‐phosphocholine), 40% cholesterol, and 6% PEG2000 DSPE (1,2‐distearoyl‐sn‐glycero‐3‐phosphoethanolamine‐*N*‐[amino(polyethylene glycol)‐2000]) (Avanti Polar Lipids, Alabaster, AL). Fluorescent liposomes contain 1–2 mol% fluorescent lipid in place of DSPC: TopFL‐PC was used for NTA, AF594 was used for flow cytometry experiments, and Liss RhodPE for IHC experiments (all from Avanti Polar Lipids). Lipid solutions were subjected to a constant stream of nitrogen gas until visibly dried, and then further dried in a vacuum chamber for 1–2 h to remove residual solvent. These dried lipid films were rehydrated with PBS (for empty liposomes) or 300 mM ammonium sulfate buffer at pH 5.5 (as a trapping agent solution for nintedanib‐loaded liposomes). The rehydrated lipid solution underwent three freeze/thaw cycles by alternating exposure to dry ice in ethanol or 65 °C water bath. The lipid solution was then extruded at 65 °C for 10.5 cycles through a 200 nm polycarbonate filter using an Avanti Mini Extruder (Avanti Polar Lipids).

At each stage after particle synthesis and modification, particle size, distribution, and polydispersity index in PBS (for empty liposomes) or DI water (for nintedanib‐loaded liposomes) were measured at 25 °C using dynamic light scattering on a Zetasizer PRO Red. Particle concentration in ultrapure DI water was measured at 25 °C using a NanoSight NS300 (both instruments by Malvern Panalytical, Malvern, UK).

TopFL‐PC fluorescent liposomes were separated from free fluorescent lipid by sepharose column chromatography, and then elution fractions representing liposomes and free fluorescent lipid were placed on a plate reader to measure fluorescence in each fraction. This was repeated following incubation in PBS and incubation in ex vivo BALF for up to 24 h. To reduce the effect of dilution, enriched BALF was used. Enriched BALF from two naive mice (combined) was collected by inserting a catheter in the trachea through which a total volume of 1 mL cold BALF buffer (0.5 mM EDTA in PBS) was sequentially instilled and then extracted three times prior to drawing out.

##### Ligand Conjugation, Characterization, and Radiolabeling of Liposomes

DBCO‐functionalized antibodies were conjugated to azide liposomes using copper‐free click chemistry as previously described by our group.^[^ ^56^, ^57^, ^58^, ^59^ ^]^ Postincubation mixtures were purified by size‐exclusion chromatography using Sepharose 4B‐Cl (GE Healthcare, Pittsburgh, PA). For radiolabeling, a tracer amount of ^125^I‐IgG‐ DBCO was conjugated on the surface of the particle at no more than 13 IgG per particle. All hazardous materials and radioactive samples were handled and disposed of according to the guidelines and policies set by the Environmental Health and Radiation Safety Department of the University of Pennsylvania. To confirm stability of fluorescent liposomes,

##### Nintedanib Remote Loading, Purification, and Dialysis Leak Study

Free trapping agent ammonium sulfate was removed with overnight dialysis (MWCO: 12‐14 kDa) in 500× volume of a 9.4% w/v sucrose solution and then an additional 2‐4 h in the morning after replacing the sucrose solution. Remote loading of nintedanib was performed at 65 °C for 20 min in a loading solution mixture composed of 25% v/v liposome solution of 60 mM lipid concentration, 5% v/v 31 mg mL^−1^ l‐Histidine in 9.4% w/v sucrose at pH 6.5, and 70% v/v 15 mg mL^−1^ nintedanib in 9.4 % w/v sucrose, followed by chilling the loading mixture on ice for 5 min. Nintedanib loading was adapted from patent # WO2019209787A1. Free trapping agent ammonium sulfate was removed with overnight dialysis (MWCO: 12‐14 kDa) in 500× volume of a 9.4% w/v sucrose solution and then an additional 2–4 h in the morning after replacing the sucrose solution. Free nintedanib that was not loaded into liposomes was purified by desalting the loading mixture with Zeba Spin Desalting Columns (7 K MWCO) at 1530 g. Liposomes were further concentrated with an Amicon filter (10–100 k MWCO, 4 mL) as needed. The drug encapsulation (i.e., loading efficiency) of the final nintedanib‐loaded liposome sample was measured with UPLC analysis (below). Drug leak was measured by adding purified liposomes inside a dialysis membrane (Spectra/Por 4 Dialysis Tubing, MWCO: 12–14 kDa, Repligen) with 500× volume of various pH buffers external to the membrane. The exterior buffer was regularly replaced with fresh buffer throughout the course of dialysis. Nintedanib concentration inside the dialysis membrane was measured at various time points using UPLC (below).

##### UPLC Method for Measuring Nintedanib Concentrations

For measuring nintedanib concentration, ultraperformance liquid chromatography was performed on an Acquity I‐Class ultraperformance liquid chromatography (UPLC) system (Waters Corp., Milford, MA, USA). Chromatography was performed using a Cortecs C18 column (1.6μ m particle size, 2.1 × 50 mm). A gradient elution program was conducted for chromatographic separation with mobile phase A (water with 0.1% trifluoroacetic acid) and mobile phase B (acetonitrile with 0.1% trifluoroacetic acid) as follows: 0–0.4 min (90–90% A), 0.4–0.8 min (90–10% A), 0.8–1.3 min (10–10% A), 1.3–1.4 min (10–90% A), and 1.4–2.6 min (90–90% A). The flow rate was 0.50 mL min^−1^ and the sample injection volume was 8 μL. The overall run time was 2.6 min and retention time for nintedanib was ≈1 min. The wavelength for UV detection was 391 nm. A stock solution of nintedanib (4 mM) and working standards were prepared in a 50/50 v/v mixture of mobile phases A and B. Liposome samples were prepared by diluting the liposomes 100‐fold into a 50/50 v/v mixture of mobile phases A and B.

A calibration curve was created by analyzing standards with known concentrations of nintedanib. The peak area was plotted against the known drug concentration and a linear regression (weighted by 1/(drug concentration)^2^) was performed. The calibration standards ranged from 0.004 to 4.23 mM. The correlation coefficient of the calibration curve (*R* ^2^) was 0.99. The precision/repeatability (as measured by coefficient of variation [CV]) was 2.4%. The accuracy ranged from 97.2% to 105.9%. The LLOQ was 0.004 mM and the ULOQ was 4.23 mM. No carryover was observed.

##### Transmission Electron Microscopy of Liposomes

Negative stain electron microscopy was performed by the Electron Microscopy Resource Lab (EMRL) at the Perelman School of Medicine, University of Pennsylvania. The images were recorded on a Gatan Oneview 4Kx4K camera. Each image was collected by exposing the sample for 4 s and a total of 100 dose fractionated images were collected at −1.5 to 2 μm under focus at 30–40 K magnification and composed in a single micrograph.

##### Intratracheal Instillation and BALF Collection

Mice were suspended vertically via the upper incisors using a surgical thread loop on a surgery board. The tongue was pulled laterally and gently extended using sterile padded forceps to visualize the vocal cords. Fifty microliter of liquid was instilled into the back of the oral cavity for inspiration. After hearing a gasp that confirms endotracheal delivery of the liquid, the nose was covered intermittently to encourage instillation. Animals were terminally anesthetized by intramuscular injection of Ketamine/Xylazine stock solution and euthanized by exsanguination. BALF was collected by inserting a catheter in the trachea through which a total volume of 2.4 mL cold BALF buffer (0.5 mM EDTA in PBS) was instilled into the airway separated into three equal volumes. For experiments separating BALF into supernatant and pellet, centrifuge settings of 2500 rpm for 10 min at 4 C were used.

##### LC–MS/MS Method for Analysis of Nintedanib in Mouse Plasma and Lung Homogenate

LC**–**MS/MS was performed on plasma and lung homogenates by the Bioanalytical Core Laboratory of Children's Hospital of Philadelphia. Nintedanib and Intedanib‐d3 (as the internal standard) were analyzed by ultraperformance liquid chromatography (UPLC) and detected using a triple quadrupole mass spectrometer (API4000).

##### NTA of BALF following In Vivo Liposome Instillation

A total volume of 50 μL of liposomes of concentration 6.66 × 10^12^ particles mL^−1^ (corresponding to 10 mg kg^−1^ lipid dose) was given to each mouse via intratracheal instillation. At designated time points up to 20 h after liposome administration, BALF was collected and analyzed. NTA was conducted with a 488 nm excitation laser and a 500 nm long‐pass filter to assess fluorescent liposomes (TopFluor PC lipid) without endogenous species. Automated analysis of fluorescence nanoparticle tracking data in Malvern Nanosight software used a uniform detection threshold of three for all samples. The same samples were immediately analyzed a second time with an open filter to assess light scattering species to track the total population of liposomes plus endogenous species in both BALF and plasma. Scattering‐based nanoparticle tracking data were analyzed in Malvern Nanosight software with a detection threshold of 5–12. For both fluorescence data and scattering data, five technical replicates were obtained for each sample and an average of those replicates was taken as representative of the size‐concentration profile for each sample. Size‐concentration profiles, size values, concentration values, and polydispersity index values presented represent averages of biological replicates (*N* = 3–6 biological replicates per time point) and movies/images of NTA data represent individual technical replicates.

##### Lung Biodistribution Study

Fifty microliter aliquots of radiolabeled liposomes of varied liposome concentration and lipid dose were administered by intratracheal instillation to C57BL/6 male mice (The Jackson Laboratory, Bar Harbor, ME). Specific concentration and dose are provided per experiment, with a range of 8E11–2E13 particles mL^−1^ and 1.25–30 mg kg^−1^ lipid. Animals were euthanized at designated times after instillation (30 min, 1, 3, 6, and 24 h after administration); blood samples were collected using 0.5 m EDTA‐coated syringes, and the organs of interest were harvested, rinsed with water, blotted dry, and weighed. Radioactivity in organs and blood were measured with a Wallac 1470 Wizard gamma counter (PerkinElmer Life and Analytical Sciences‐Wallac Oy, Turku, Finland). The total instilled dose was measured using liposome samples before instillation, and the radioactivity data of the ^125^I measurements were used to calculate the tissue biodistribution percent instilled dose. Percent instilled dose (%ID) was calculated by dividing the sum of radioactivity detected in collected sites (lung, blood (scaled to 2 mL), liver, spleen, stomach, intestine, and oropharynx) divided by the total instilled dose.

##### Single‐Cell Preparation of Lung Homogenate and Flow Cytometry Analysis

Single‐cell suspensions were prepared from male C57BL/6 mouse lungs for flow cytometry. Fluorescent liposomes were administered through intratracheal instillation delivery. Animals were sacrificed and their lungs were harvested after perfusion of lungs via the right ventricle with ≈10 mL of PBS at a constant pressure of 25 cm H_2_O. Following manual disaggregation, single‐cell preparation of whole lung was prepared for fluorescent antibody staining for each cell type of interest. APC‐anti‐CD45, AF700‐anti‐Ly6G, PE‐cy7‐anti‐CD64, APC‐cy7‐anti‐EPCAM, and PE‐cy7‐anti‐CD31 antibodies (all from BioLegend) were used for staining leukocytes, neutrophils, macrophages/monocytes, epithelial cells, and endothelial cells, respectively. Stained cells were then fixed in 4% PFA before resuspension in FACS buffer for storage no more than 24 h prior to analysis. Flow cytometry was performed on a BD LSRFortessa Cell Analyzer (BD Biosciences). Controls with no stain were obtained from naive and PBS‐instilled mice; FMO controls were used for CD31, EpCAM, Ly6G, and CD64 when applicable. Data were analyzed with FlowJo software (FlowJo LLC). Gates were determined by gating for singlets, and then selecting cell populations that had ≤1% overlap with unstained controls and FMO controls; gating strategy is shown in Figure S6, Supporting Information.

##### IHC

Mice were given 50μ L Liss Rhod PE‐labeled liposomes via intratracheal instillation 30 min prior to sacrificing the animals. Ten minutes prior to sacrifice, animals were injected with anti‐PECAM monoclonal antibodies labeled with AF647 (clone MEC13.3). Lungs were harvested and then immediately immersed in OCT and frozen in liquid N_2_. Frozen lung tissue was cut using Cryostat at a thickness of 20 μm and then washed with PBS and mounted with ProLong Gold antifade reagent with DAPI (Invitrogen). Microscopy studies were performed on a confocal laser scanning microscope Leica TCS‐SP8 (Leica, Germany) using HC PL APO CS2 63×/1.40 oil objective and 488/552/638 lasers. Image analysis was performed using Fiji ImageJ2.

##### Toxicity Characterization: CBC, Histology, BAL Leukocyte, and Protein Characterization

All samples used for the toxicity study were collected 24 h postintratracheal instillation of liposomes or control solution. Blood was drawn into 0.5M EDTA‐coated tubes and a CBC was measured, including white blood cells, platelets, RBC, hemoglobin (Hgb), and hematocrit (Hct) using an Abaxis VetScan HM5. Whole lungs were inflated and fixed with neutral buffered 10% formalin. Paraffin‐embedded 5 μm lung sections were stained with H&E by the Pathology Core Laboratory of Children's Hospital of Philadelphia. BAL was collected as previously described, fixed, and stained with APC‐anti‐CD45 antibody. Flow cytometry of BAL cells was performed on an Accuri C6 (BD Biosciences) and analysis was done using FCS express. Data were gated to exclude debris and doublets.

##### Pharmacokinetics Modeling and Simulation

Noncompartmental analysis was performed using the built‐in module in GastroPlus (version 9.8, Simulation Plus, INC) based on our in vivo LC/MS data for oral nintedanib, instilled free nintedanib, and instilled nintedanib‐loaded liposomes. Key PK parameters for blood and lungs were obtained by direct curve‐stripping (concentration vs time): half‐life from the log‐linear terminal slope and AUC from the linear trapezoidal rule.

##### Animal Protocols

All animal studies were carried out in strict accordance with the Guide for the Care and Use of Laboratory Animals as adopted by the National Institutes of Health and approved by the University of Pennsylvania Institutional Animal Care and Use Committee (#805 696). All animal experiments used male C57BL/6 J mice, 6–8 weeks old, purchased from Jackson Laboratories. Mice were maintained at 20–25 °C, 50% ± 20% humidity, and on a 12/12 h dark/light cycle with food and water ad libitum.

##### Statistical Analysis

Data for all experiments were recorded using Excel and analyzed using Prism. For all animal experiments, *N* ≥ 3 mice with the exception of flow cytometry and IHC where *N* = 2 mice (Figure 5). No data points were excluded from analysis. Error bars indicate the standard error of the mean throughout. Statistical significance was calculated using a one‐way ANOVA with Tukey's post hoc test for multiple comparisons. A significance level of 0.05 was used to determine statistically significant differences between groups.

## Conflict of Interest

The authors declare no conflict of interest.

## Supporting information

Supplementary Material

## Data Availability

The data that support the findings of this study are available from the corresponding author upon reasonable request.
