# Supplementary material for: Mechanisms by Which Liposomes Improve Inhaled Drug Delivery for Alveolar Diseases
Source: Adv Nanobiomed Res. 2023 Jan 27;3(3):2200106. doi: 10.1002/anbr.202200106 (PMC10231510; doi:10.1002/anbr.202200106)
Supplement: Supplementary file 1 — Supplementary Material [file ANBR-3-2200106-s001.pdf]

Supplemental Figures

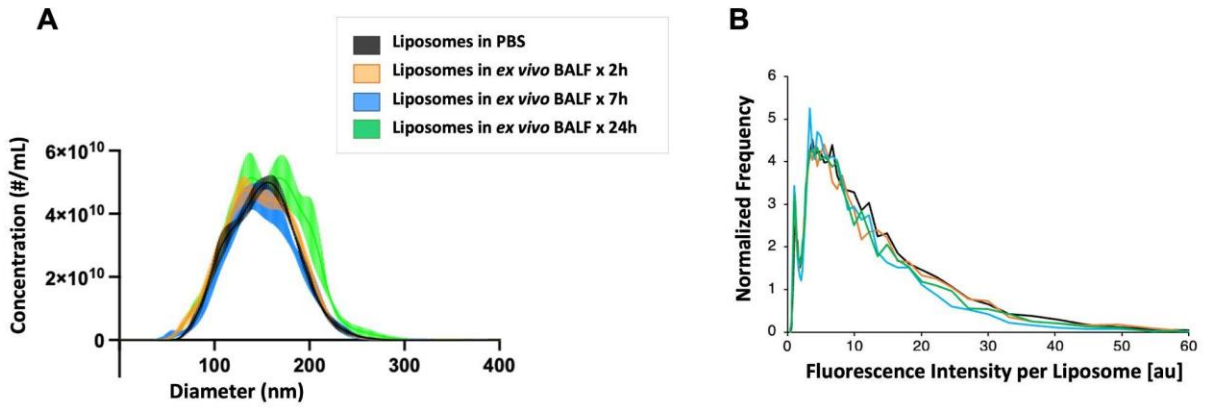

Supplemental Figure 1. Assessment of fluorescent liposome stability before liposome incubation in BALF and after 2, 7, or 24 hours incubation in *ex vivo* BALF. (A) Assessment of fluorescent liposome size distributions before and after incubation in *ex vivo* BALF up to 24 hours. The size distributions here show the absolute concentrations for each condition, indicating no loss of fluorescent liposome concentration during incubation in BALF. The data here were used to make the normalized size distributions shown in Figure 1G. **(B)** Liposome fluorescence intensity distributions vs time. Per liposome fluorescence intensity was determined in NTA experiments. Normalized distributions of per liposome fluorescence intensity are depicted.

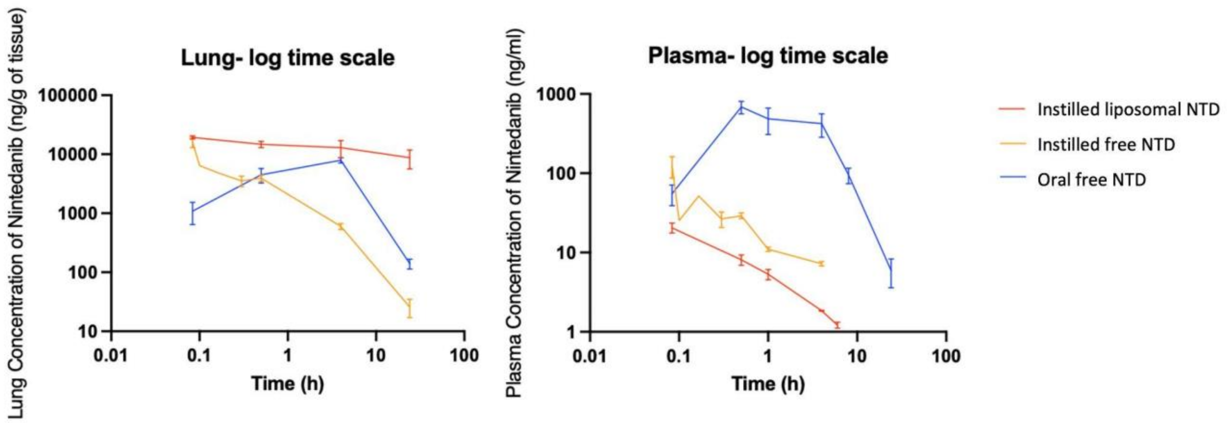

Supplemental Figure 2. Inhaled nintedanib-loaded liposomes confer massive increases in lung half-life and AUC compared to inhaled or oral free nintedanib. Mice were given one of three nintedanib formulations, shown in Figure 2A (intratracheal instillation of liposome-loaded nintedanib (red), intratracheal instillation of free nintedanib (orange), or oral gavage of free nintedanib (blue)). Lung and plasma were harvested at time points up to 24 hours, then nintedanib concentration was measured by LC/MS. Nintedanib concentrations over 24

hours are shown on a log-scale of drug concentration. N=3 (exception: minor errors in collection time for instilled free NTD between 15 min and 1 hour were reported individually rather than grouped by time point and shown without error bars); error bars = SEM.

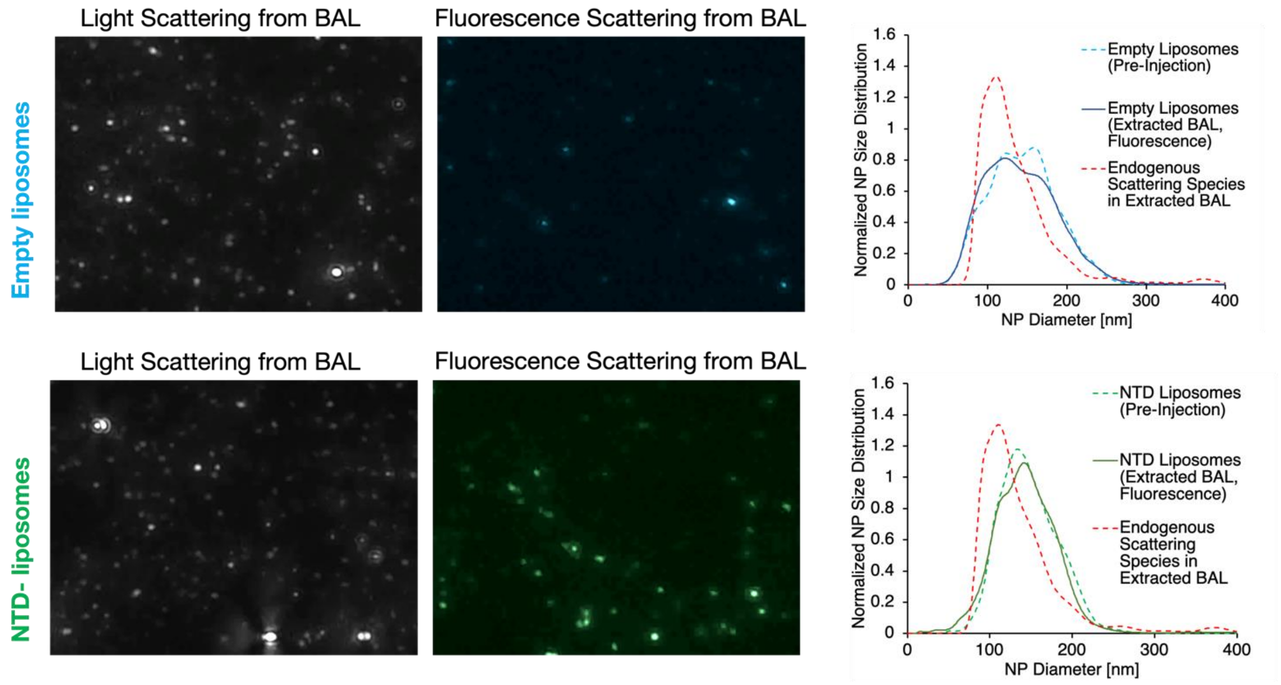

Supplemental Figure 3. Additional primary data demonstrating that liposomes remain intact and do not lyse or aggregate after exposure to mucus and surfactant *in vivo.* Mice were given 2.5mg/kg lipid of fluorescent liposomes or nintedanib- loaded fluorescent liposomes via intratracheal instillation, then bronchoalveolar lavage (BALF) fluid was harvested and analyzed by nanoparticle tracking analysis (NTA). **(Top Row)** Primary data showing NTA of endogenous particles in BAL by light scattering (left) and liposomes in BALF by fluorescence (middle). Right panel shows a normalized histogram of liposomes in their native form before being given to mice (dotted green line, fluorescence measurement), compared to liposomes extracted in BAL fluid 15 minutes after inhaled delivery (solid green line, fluorescence measurement), compared to endogenous particles + liposomes extracted in BALF fluid after inhaled delivery (dotted red line). **(Bottom Row)** The data shown here is a replica of what's shown in the top row, except that the liposomes here are all loaded with nintedanib (NTD), while the data in the top row was all for empty liposomes.

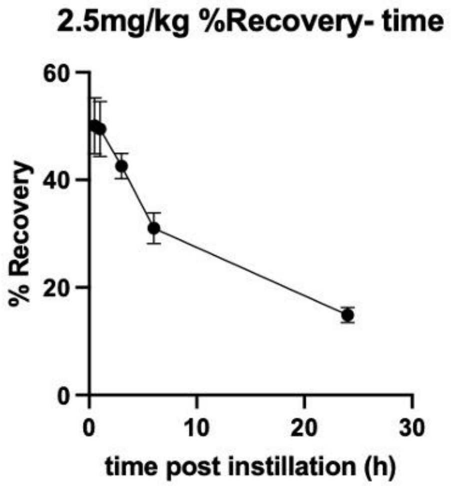

Supplemental Figure 4. Percent recovery for lung biodistribution studies in Figure 4b. Percent recovery is defined as the sum of collected organs (lung, liver, spleen, stomach, colon (only at 24h), oropharynx, blood) divided by total administered dose. Total dose was independently measured by gamma counter prior to instillation. Percent recovery for initial lipid dose of 2.5mg/kg is shown.

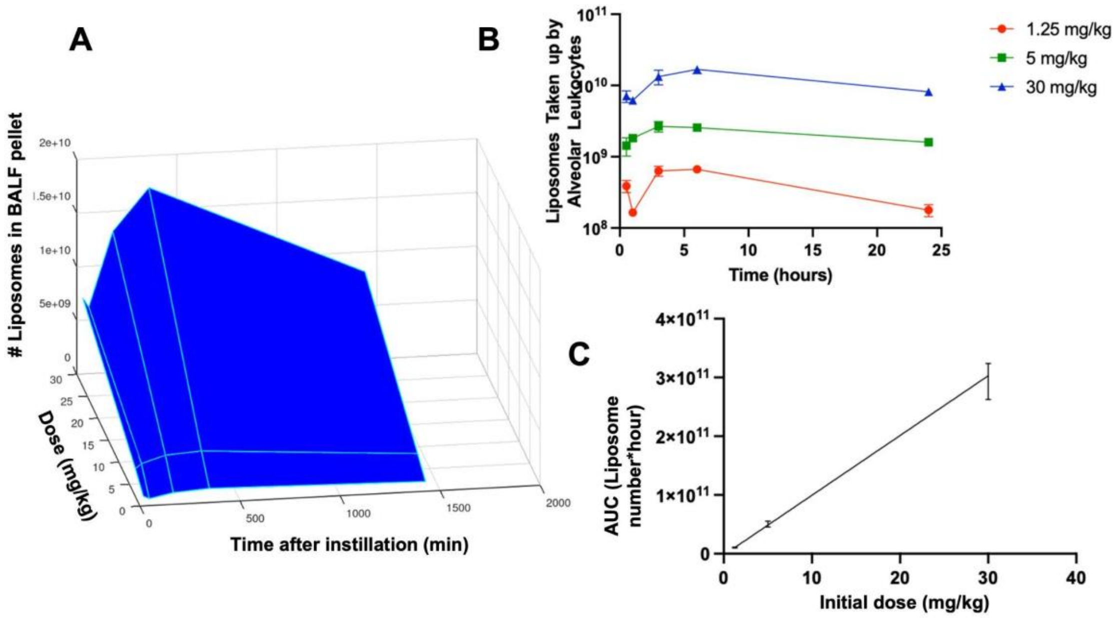

Supplemental Figure 5. Alveolar cell compartment (BALF pellet) shows no saturation. (A) 3D representation of the uptake kinetics of alveolar leukocytes. Peak uptake of liposomes by alveolar leukocytes is seen at 6h post instillation followed by moderate decline over 24h. This pattern is maintained at all doses. **(B)** PK curves for the number of liposomes taken up by alveolar leukocytes (BALF pellet) over time; data shown for three separate initial doses. N=3 mice per dose and timepoint. From here, AUC was calculated for each dose. **(C)** AUC for each of three doses was calculated from (B) and is shown here as a linear regression with R-squared value of 0.96. N=3, error bars = SEM.

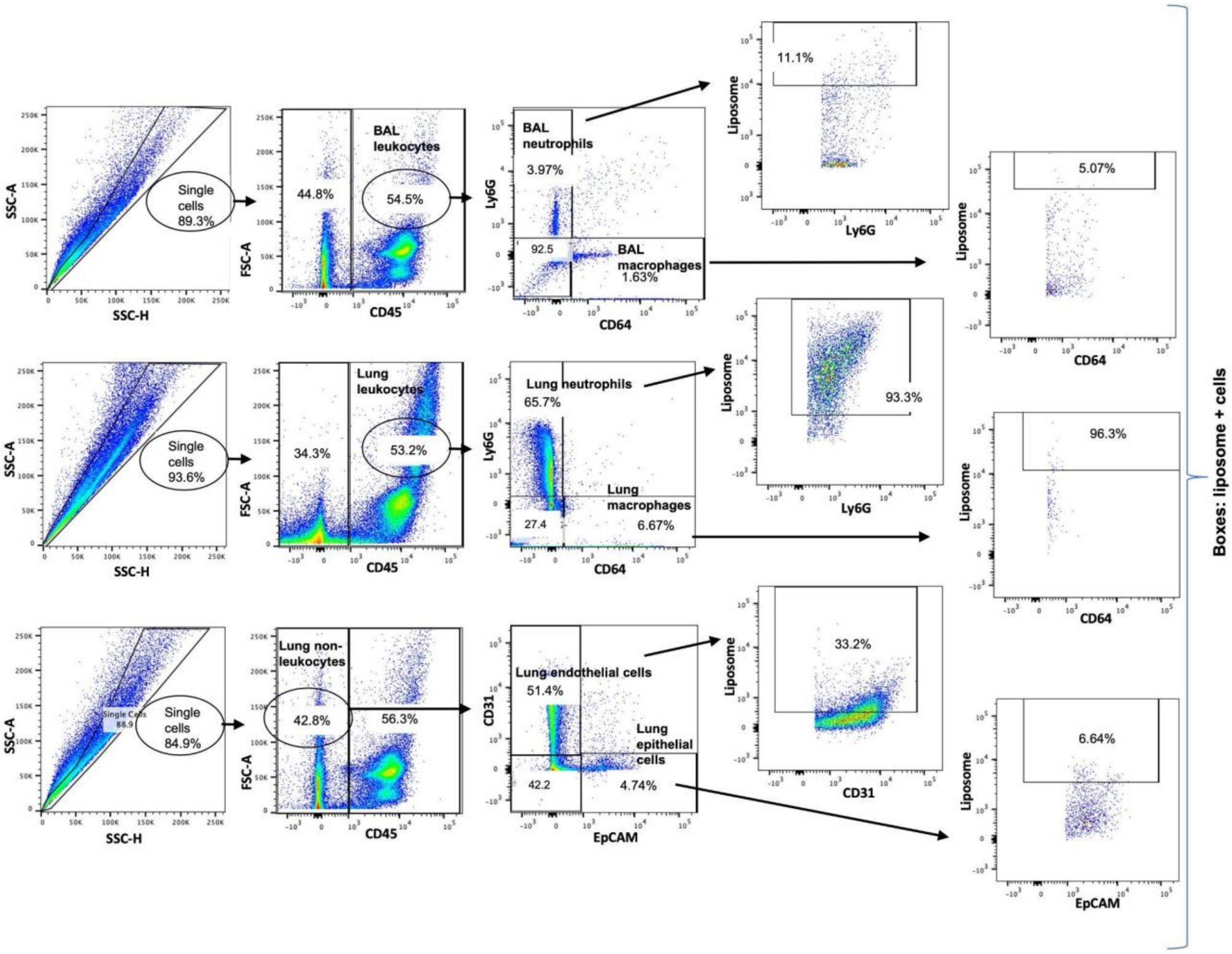

Supplemental Figure 6. Gating schemes for flow cytometry data in Figure 5. Mice were given fluorescent liposomes (empty and drug- loaded) via intratracheal instillation, then at 20h whole lung was analyzed. Prior to harvest and preparation of single-cell suspension for flow cytometry, BAL and perfusion were performed. **(Top row)** BALF gating scheme; this represents alveolar (airway) leukocytes. **(Middle row)** Gating scheme for parenchymal leukocytes- white blood cells within the interstitial space or firmly adhered to the endothelium. **(Bottom row)** Gating scheme for all non-leukocyte parenchymal cells.

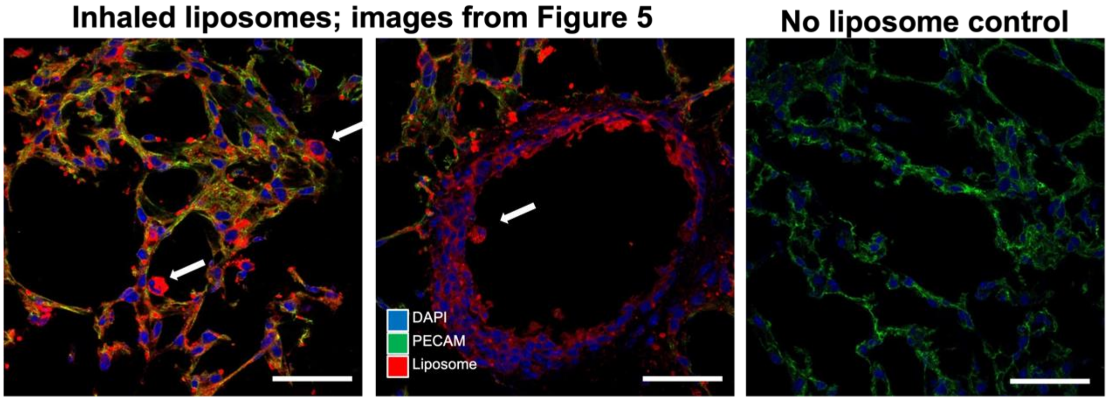

Supplemental Figure 7. Immunohistochemistry control for microscopy data in Figure 5. Mice were given fluorescent liposomes via intratracheal instillation, then at 20h whole lung was analyzed. Immunohistochemistry demonstrates liposomes (red) in all layers of lung tissue in both alveolated tissue (**left panel**) and medium- sized airways (**middle panel**). Control mouse that was not given intratracheal liposomes (**right panel).** Scale bar = 50um.

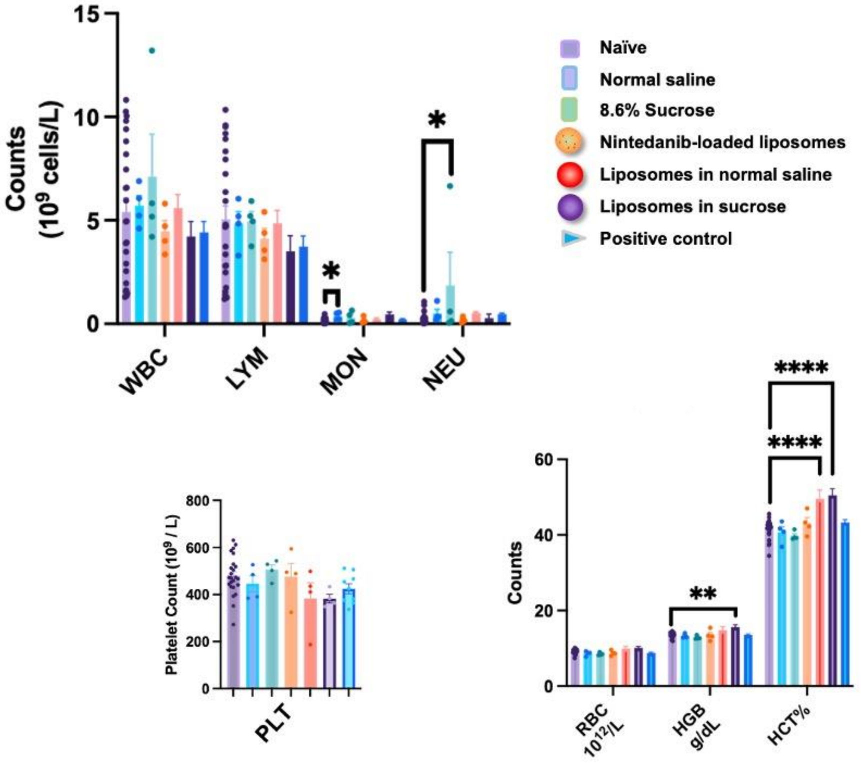

Supplemental Figure 8. Additional data for Nintedanib-loaded liposomes demonstrating no toxicity. Toxicity studies at 24h. (G) Naive murine lung is compared to intratracheal instillation of buffer, empty liposomes (2.5mg/kg lipid), nintedanib- loaded liposomes (0.5mg/kg drug and 0.73mg/kg lipid) in sucrose, or nebulized LPS (a positive control for injury), showing no change in white blood cell (WBC) count including lymphocytes, monocytes, or neutrophils; no change in platelet (PLT) count, and no change in red blood cell (RBC) count. There is a small but significant change in hematocrit likely related to changes in intravascular volume.

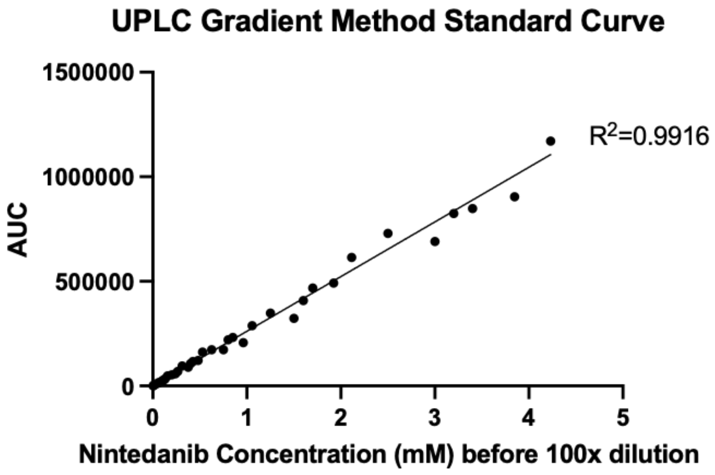

Supplemental Figure 9. Calibration Curve for Nintedanib Gradient Method. A calibration curve was created by analyzing samples with known concentrations of nintedanib. The peak area was plotted against the known drug concentration and a linear regression (weighted by 1/(drug concentration)^2) was performed. The calibration standards ranged from 0.004mM to 4.23mM (all samples analyzed were in the range of 0.2 mM to 3 mM nintedanib concentration, which is well within the dynamic range of measurements).
